# Stress Biomarkers in Young Adult University Students Before, During, and After the COVID-19 Pandemic: A Longitudinal Cohort Study

**DOI:** 10.7759/cureus.97328

**Published:** 2025-11-20

**Authors:** Ioannis Chaniotakis, Aikaterini Anetaki, Constantine Anetakis, Eleftherios Panteris, Sotirios Varlamis, Petros Skepastianos, Maria Chatzidimitriou, Matthaios Bobos, Vasileios Papaliagkas, Stella Mitka

**Affiliations:** 1 Department of Biomedical Sciences, International Hellenic University, Thessaloniki, GRC; 2 Neonatology/Neonatal Intensive Care Unit (NICU), University General Hospital of Heraklion, University of Crete, Heraklion, GRC

**Keywords:** cortisol, covid-19, dopamine, hypothalamic-pituitary-adrenal (hpa) axis, post-covid stress, serotonin, stress biomarkers, stress-related disease

## Abstract

Introduction: Although life expectancy has increased, modern life has intensified stress factors, affecting both physiology and mental health. The COVID-19 pandemic has been a prolonged, stressful event with significant implications for young adults, especially students. The hypothalamic-pituitary-adrenal (HPA) axis and related hormonal markers such as cortisol and free cortisol are important biomarkers of the body’s response to stress, while dopamine and serotonin are neurochemical biomarkers of mood and pleasure.

Aim: This study aims to assess and correlate hormonal stress biomarkers and neurotransmitters with behavioral factors over time, to investigate the effects of the pandemic on the neurobiology and behavior of young adult university students.

Materials and methods: This is a prospective non-interventional cohort study that examines the levels of key stress hormones (cortisol, free cortisol), mood and pleasure neurotransmitters (serotonin, dopamine), as well as behavioral and demographic parameters (screen exposure, BMI, physical activity) in students from health faculties of various universities in northern Greece, aged 18-25 years, in three time periods: before (2019-2020), during (2020-2022), and after the pandemic (2023-2025). The biomarkers were measured in serum samples, using immunoenzymatic methods for dopamine and serotonin and immunochemiluminescence for cortisol and free cortisol.

Results: The findings showed a significant increase in total and free cortisol levels and mobile phone use during and after the pandemic, as well as a sustained decrease in serotonin and dopamine. In contrast, regular physical exercise was associated with a more favorable hormonal profile, regardless of the epoch.

Conclusions: The findings highlight the impact of long-term stressors on the biochemical balance of stress-hormones and neurotransmitters and the long-term biological and behavioral effects of the pandemic on university students, with potential consequences for their future somatic and mental health. Physical exercise emerges as an important protective factor against the effects of chronic stress, as does timely mental health monitoring and support.

## Introduction

During the coronavirus disease 2019 (COVID-19) era, the daily life of young adult university students shifted with campus closures, remote learning, reduced physical activity, and intensified digital use. These changes created sustained biological demands on stress-regulatory systems. A biology-first lens focuses on the hypothalamic-pituitary-adrenal (HPA) axis and the sympathetic-adrenomedullary system, whose endocrine outputs provide objective indices of allostatic load [1-3].

Cortisol, the principal glucocorticoid end-product of the HPA axis, exhibits a diurnal profile with a cortisol-awakening response and a declining daytime slope. Prolonged psychosocial challenge can recalibrate this system, yielding altered basal levels, flattened diurnal slopes, or increased overall exposure, patterns consistent with HPA dysregulation [3-5]. Dehydroepiandrosterone sulfate (DHEA-S) partially counterbalances glucocorticoid actions; consequently, the cortisol/DHEA-S ratio serves as a systems-level marker of anabolic-catabolic balance under chronic demand [3,4]. In parallel, sympathetic activation modulates rapid cardiovascular and metabolic responses via catecholamines and may remain tonically elevated under persistent vigilance, sedentary routines, and irregular schedules that frequently accompany remote formats and heavy screen exposure [3,6].

University students are a suitable population for studying stress biology. Before the pandemic, academic pressure, transitional roles, and screen-centric study habits were already linked to measurable changes in endocrine and immune markers [7-10]. Experimental and quasi-experimental work further shows that standardized psychosocial stressors affect diurnal cortisol and related biomarkers, underscoring the biological sensitivity of these endpoints in young adults [11]. These observations support targeting hormone profiles, including cortisol, DHEA-S, and catecholamine-linked indices, to quantify biological stress across discrete pandemic epochs while accounting for behavioral covariates such as daily digital use, body mass index (BMI), physical activity, and sleep timing [1-3].

In sum, an endocrine-centered approach grounded in HPA and sympathetic physiology offers a rigorous framework to examine whether pandemic-era behavioral shifts corresponded to changes in stress-hormone biology in young adults. This focus aligns with established psychobiological models and with prior evidence in student samples, providing a rationale for hypothesis-driven analyses of stress-hormone levels before, during, and after the COVID-19 period [3,7,8,11].

## Materials and methods

Participants and ethical considerations

This was a non‑interventional, prospective cohort study of healthy university students in Northern Greece. Recruitment and data collection were conducted at the Clinical Chemistry Laboratory, Department of Biomedical Sciences, International Hellenic University (IHU). The study received approval from the IHU Research Ethics Committee (Session 16/06‑06‑2025; Ref. No. 123/2025) and adhered to the Declaration of Helsinki. All participants provided written informed consent for the use of anonymized data. Participation was voluntary, and students could withdraw at any time without consequences; data collected up to the point of withdrawal could be retained for analysis unless otherwise requested.

Data were recorded in de‑identified electronic files under the General Data Protection Regulation (GDPR) principles to prevent linkage to personal identifiers. Personal data were protected under the EU Regulation 2016/679 (GDPR) and Greek Law 4624/2019. Only aggregated results are reported, and no individual participant can be identified.

Study design and setting

We enrolled 100 students aged 18 to 24 years from health‑related schools, including the Department of Biomedical Sciences IHU, the Departments of Physiotherapy and Nursing IHU, the School of Medicine and the Department of Molecular Diagnostics at Democritus University of Thrace, and the Schools of Medicine and Biology at Aristotle University of Thessaloniki. Sampling occurred outside examination periods and exclusively in May, in the morning, after weekend rest, under resting conditions. Each participant contributed three serum samples representing three epochs: pre‑pandemic (May 2018 and May 2019), during restrictions (May 2020 through May 2022), and post‑pandemic (May 2024 through May 2025).

Exclusion criteria were any acute or chronic disease, intercurrent illness at sampling, any medication use, or acute stress states such as exhaustion or recent surgery.

Data collection and blood sampling

Venipunctures and anthropometry were performed at the Clinical Chemistry Laboratory, Department of Biomedical Sciences, IHU. During restriction periods, additional blood draws were carried out at accredited local diagnostic laboratories following the same protocol; samples and weight measurements were provided by participants with written or verbal consent.

Blood was collected without an anticoagulant. After approximately 60 minutes at room temperature, tubes were centrifuged for 20 minutes at 1000×g. Serum was aliquoted into three pyrogen‑free tubes. Samples analyzed within seven days were stored at 2-8°C; otherwise, aliquots were frozen at −20°C for up to one month or −80°C for up to three months. Multiple freeze-thaw cycles were avoided. Hemolyzed or lipemic sera were not used. Before analysis, frozen aliquots were fully thawed and gently mixed without vortexing.

Participants answered a brief questionnaire covering age, sex, height, weight, BMI, physical activity, medical history, and medications. BMI was calculated as weight in kilograms divided by height in meters squared.

Laboratory assays

The following biomarkers were quantified at each epoch: total cortisol, free cortisol, dopamine, and serotonin. All assays were performed at the Clinical Chemistry Laboratory, Department of Biomedical Sciences, IHU, following manufacturers’ protocols and laboratory quality‑control procedures.

Cortisol

Total and free cortisol concentrations were measured on an Elecsys immunoanalyzer (Roche Diagnostics, Mannheim, Germany) using electrochemiluminescence immunoassays.

Dopamine and Serotonin

Analytes were measured with competitive enzyme-linked immunosorbent assay (ELISA) kits (Thermo Fisher Scientific Inc., Bender MedSystems GmbH, Vienna, Austria).

Data structure and pre‑processing

Numeric fields were standardized to two decimal points. Sex was harmonized as male and female. Physical activity was coded 0 for "No" and 1 for "Yes." Missing values were handled listwise within each analysis. For repeated‑measures analyses, data were reshaped to long format with one row per participant per time point. The pre‑pandemic sample served as the reference time point for modeling.

Statistical analysis

Descriptive statistics are reported overall and by sex. Categorical variables are summarized as a number (percentage). Continuous variables are summarized as mean ± standard deviation when approximately normal, or median (interquartile range) otherwise. Sex comparisons used Mann-Whitney U tests for non‑normal continuous variables and χ² tests for categorical variables.

Longitudinal comparisons across three time points used the Friedman test for non‑normal repeated measures, with effect size expressed as Kendall’s $W = \chi^2 / [N \cdot (k - 1)]$. For repeated binary outcomes such as physical activity, Cochran’s Q test was applied.

Primary inferential analyses for biomarkers concentrations used linear mixed‑effects models with random intercepts for participants: Y_it = β₀ + β₁·I[During] + β₂·I[Post] + β₃·I[Female] + β₄·BMI_it + β₅·PhysicalActivity_it + β₆·ScreenTime_it + u₀i + ε_it, with u₀i ~ N(0, σᵤ²) and ε_it ~ N(0, σ²). Time was categorical with "pre" as the reference. Covariates were time‑varying where available. Models were estimated by maximum likelihood with L-BFGS (limited-memory Broyden-Fletcher-Goldfarb-Shanno) optimization. The omnibus effect of time was tested using likelihood ratio tests with two degrees of freedom. Planned pairwise contrasts (During-Pre, Post-Pre, Post-During) were evaluated with Wald tests and 95% confidence intervals. Model diagnostics included inspection of residual distributions and variance homogeneity.

Software

Analyses were performed in Python 3.11.8 (pandas 1.5.3, numpy 1.24.0, SciPy 1.14.1, statsmodels 0.13.5) (Python Software Foundation, Wilmington, Delaware) and IBM SPSS Statistics version 26 (IBM Corp., Armonk, NY) for descriptive summaries and figures.

## Results

Demographic characteristics

A total of 100 university students from institutions in Northern Greece participated. The sex distribution was 56% versus 44% without a statistically significant difference between groups (p = 0.230). Baseline age was comparable across groups, with a median of 20 years (19-22) overall. Baseline anthropometry and behaviors are summarized in Table 1.

**Table 1:** Demographics & baseline characteristics. Data are presented as n (%) or median (Q1-Q3). P-values are from χ² tests for categorical variables and Mann-Whitney U tests for continuous variables. Two-sided significance was set at p < 0.001. * P-value from χ² test.

Variable	Men	Women	Total	P-value (Mann–Whitney)
Age (years)	20.00 (19.00-22.00)	20.00 (19.00-22.00)	20.00 (19.00-22.00)	0.821
Sex	56 (56.0%)	44 (44.0%)	100 (100.0%)	0.230*
Baseline BMI	25.50 (24.95-27.70)	24.40 (20.98-25.93)	25.10 (23.68-27.70)	<0.001
Baseline multimedia use (hours)	5.00 (2.00-6.00)	5.00 (2.00-6.00)	5.00 (2.00-6.00)	0.623
Baseline exercise (Yes)	19 (33.9%)	22 (50.0%)	41 (41.0%)	0.156*

BMI at baseline was higher in men relative to women (median = 25.50 (24.95-27.70) vs. 24.40 (20.98-25.93); overall median = 25.10 (23.68-27.70); p < 0.001). Baseline daily screen time showed a median of five hours per day (2-6) in both groups, with no between-group difference (p = 0.623). At baseline, 41 participants (41.0%) reported engaging in regular physical activity; proportions did not differ significantly between groups (p = 0.156). Full details are provided in Table 1.

Descriptive summaries across the three predefined epochs (pre-pandemic, during restrictions, and post-pandemic) are shown in Table 2. BMI exhibited stable medians over time (pre = 25.10 (23.68-27.70), during = 25.45 (24.08-27.70), post = 25.50 (24.08-27.92)), with a non-significant Friedman test (χ² = 4.53, df = 2, p = 0.104; Kendall’s W = 0.023; N = 100 complete). Daily screen time increased across epochs from a median of five hours (2-6) pre-pandemic to six hours (5-6) during restrictions and eight hours (6-10) post-pandemic, with a significant Friedman test (χ² = 163.90, df = 2, p < 0.001; Kendall’s W = 0.819; N = 100 complete). The proportion reporting regular physical activity was stable by Cochran’s Q (41% pre, 40.8% during, 40% post; p = 0.223).

**Table 2:** Descriptive summaries across the three predefined epochs: pre-pandemic, during restrictions, and post-pandemic. Data are presented as percentages (%) or median (Q1-Q3). P-values are from longitudinal comparisons across three time points. The Friedman test was used for non‑normal repeated measures, with effect size expressed as Kendall’s W. For repeated binary outcomes, Cochran’s Q test was applied. Two-sided significance was set at p < 0.001. * Cochran’s Q test for repeated binary outcome.

Variable	Pre	During	Post	Friedman χ² (df = 2)	p	Kendall’s W	N (complete)
BMI (kg/m²)	25.10 (23.68-27.70)	25.45 (24.08-27.70)	25.50 (24.08-27.92)	4.53	0.104	0.023	100
Screen time (h/day)	5.00 (2.00-6.00)	6.00 (5.00-6.00)	8.00 (6.00-10.00)	163.90	<0.001	0.819	100
Physical activity (Yes)	41%	40.8%	40%	–	p = 0.223*	–	–

Stress-related biomarkers

Across 100 students, cortisol rose with the pandemic and did not return to baseline. Total cortisol was higher during vs. pre (β = 3.71, standard error (SE) = 0.20, p < 0.001) and post vs. pre (β = 4.23, SE = 0.30, p < 0.001), with no post-during difference. Free cortisol followed the same pattern (pre-during β = 0.41, p < 0.001; pre-post β = 0.49, p < 0.001). Screen time did not relate to total cortisol but was positively associated with free cortisol (β = 0.07 per hour, p < 0.001). Exercise did not affect total cortisol, yet was linked to lower free cortisol (β = −0.32, p = 0.01). Higher BMI tracked higher total cortisol (β = 0.15, p = 0.02).

Monoamines moved in the opposite direction. Dopamine fell during vs. pre (β = −11.43, p < 0.001) and declined further post (β = −30.87, p < 0.001). More screen time aligned with lower dopamine (β = −0.40 per hour, p = 0.05), while exercise aligned with higher dopamine (β = 3.79, p < 0.001). Serotonin also dropped during (β = −82.63, p < 0.001) and post (β = −151.39, p < 0.001). Women had higher serotonin than men (β = 17.33, p < 0.001). Exercise and BMI were positive for serotonin (β = 40.92, p < 0.001, and β = 1.40, p = 0.03). Screen time was null. In sum, HPA output increased across the pandemic epochs while reward- and mood-related monoamines decreased, with exercise marking a more favorable biomarker profile.

**Figure 1:**
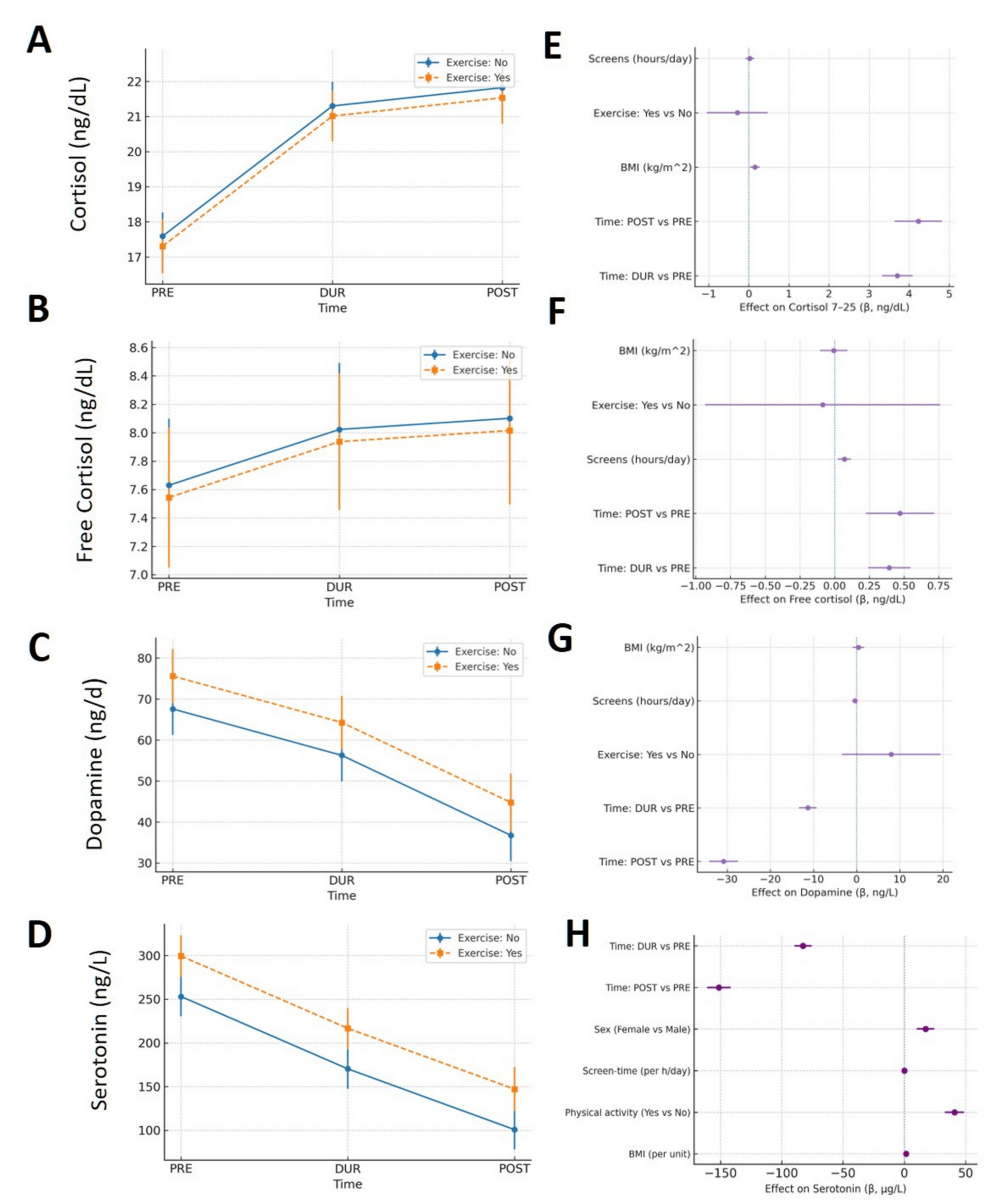
Biomarker trajectories across time points (PRE, DUR, POST) and adjusted associations. (A) Cortisol, (B) free cortisol, (C) dopamine, and (D) serotonin. Points depict mean concentration with 95% CI, stratified by self-reported regular physical activity (Yes/No). (E-H) Forest plots of adjusted mixed-effects regression coefficients (β) for each biomarker, with 95% CI for the corresponding biomarkers: covariates include time (DUR vs. PRE, POST vs. PRE), exercise (Yes vs. No), screen time (hours/day), BMI (kg/m²), and sex, where shown; random intercept for participant. Positive β indicates higher biomarker concentration. Two-sided significance set at p < 0.001. Data in (A-D) are mean (95% CI). Regression estimates in (E-H) are β (95% CI) from linear mixed-effects models; p-values are two-sided with p < 0.001.

When the post-during contrasts were explicitly tested, no further increase was observed for either total or free cortisol, confirming a plateau of HPA activation after the restriction period. In contrast, dopamine and serotonin continued to decline significantly from during to post (β = −19.44, p < 0.001 and β = −68.76, p < 0.001, respectively), indicating a cumulative reduction in monoaminergic tone beyond the acute phase. These findings suggest that while cortisol levels stabilized after the peak stress phase, neurochemical recovery of reward and mood pathways lagged behind, consistent with delayed normalization of central neurotransmitter systems following chronic stress exposure. Table 3 has all the relevant details. Figure 1 has all the biomarkers vs. time and the regression coefficients for all biomarkers and their respective mixed models.

**Table 3:** Mixed-effects model fixed effects for stress biomarkers. Reference level = Pre period. Random intercept per participant. Models adjust for sex, BMI, physical activity, and screen time (as applicable). β and standard error (SE) are unstandardized; 95% CIs computed as β ± 1.96 × SE. Two-sided significance was set at p < 0.001.

Biomarker	Effect	β	SE	95% CI	p
Total cortisol (ng/dL)	Time: During vs. Pre	3.71	0.20	3.32, 4.10	<0.001
	Time: Post vs. Pre	4.23	0.30	3.64, 4.82	<0.001
	Time: Post vs. During	0.52	0.25	0.04, 1.00	0.033
	Screen time (per h/day)	0.02	0.06	−0.10, 0.14	0.66
	Physical activity (Yes vs. No)	−0.29	0.39	−1.05, 0.47	0.46
	BMI (per unit)	0.15	0.06	0.03, 0.27	0.02
Free cortisol (ng/dL)	Time: During vs. Pre	0.41	0.07	0.27, 0.55	<0.001
	Time: Post vs. Pre	0.49	0.11	0.27, 0.71	<0.001
	Time: Post vs. During	0.08	0.09	−0.10, 0.26	0.40
	Screen time (per h/day)	0.07	0.02	0.03, 0.11	<0.001
	Physical activity (Yes vs. No)	−0.32	0.12	−0.56, −0.08	0.01
	BMI (per unit)	−0.02	0.02	−0.06, 0.02	0.33
Dopamine (ng/dL)	Time: During vs. Pre	−11.43	0.92	−13.23, −9.63	<0.001
	Time: Post vs. Pre	−30.87	1.23	−33.28, −28.46	<0.001
	Time: Post vs. During	−19.44	1.08	−21.55, −17.33	<0.001
	Screen time (per h/day)	−0.40	0.20	−0.79, −0.01	0.05
	Physical activity (Yes vs. No)	3.79	0.92	1.99, 5.59	<0.001
	BMI (per unit)	0.40	0.14	0.13, 0.67	0.004
Serotonin (μg/L)	Time: During vs. Pre	−82.63	3.55	−89.59, −75.67	<0.001
	Time: Post vs. Pre	−151.39	4.92	−161.03, −141.75	<0.001
	Time: Post vs. During	−68.76	4.30	−77.18, −60.34	<0.001
	Sex (Female vs. Male)	17.33	3.67	10.14, 24.52	<0.001
	Screen time (per h/day)	0.02	0.84	−1.63, 1.67	0.98
	Physical activity (Yes vs. No)	40.92	4.03	33.02, 48.82	<0.001
	BMI (per unit)	1.40	0.63	0.17, 2.63	0.03

## Discussion

This study demonstrates that the COVID-19 pandemic led to significant and prolonged neuroendocrine and neurochemical modifications in young adult university student participants, with an emphasis on changes in cortisol, dopamine, and serotonin. Total and free cortisol levels increased significantly during the pandemic and did not return to pre-crisis values in the post-pandemic phase, indicating prolonged activation of the HPA axis [1,4,12,13].

Similar findings have been reported in previous studies, where increased cortisol levels were recorded in saliva or hair samples from students during and after the period of restrictive measures [14,15]. However, in this study, cortisol was not measured in saliva and hair, but instead, free cortisol was measured in the participants' serum. Free cortisol in serum is comparable and consistent with cortisol measurements in saliva presented in other published studies. The measurement of free cortisol in serum is a reliable indicator of biologically active cortisol in the body. However, due to technical difficulties and cost, the use of non-invasive samples, such as saliva, has gained wide acceptance in research and clinical practice. The literature consistently supports that salivary cortisol accurately reflects unbound serum cortisol. More specifically, studies have shown very high correlation coefficients (r > 0.85) between the two measurements [16,17]. HPA axis hyperarousal is associated with increased cortisol production under chronic stress, which in the long term can lead to disturbances in metabolism, mood, and cognitive function [3,12,13].

The use of digital devices (screen time) showed no correlation with total cortisol, but showed a positive correlation with free cortisol levels, suggesting that increased exposure to digital stimuli may directly activate the stress response [18]. This finding is consistent with recent interventional studies that have shown that limiting screen use leads to improved mental health indicators and reduced stress biomarkers [19,20].

In contrast, physical exercise showed a significant negative correlation with free cortisol, which supports its role as a modifiable factor regulating the HPA axis [21,22]. This effect appears to be related to the ability of exercise to enhance feedback mechanisms and increase the sensitivity of glucocorticoid receptors [5,22].

An important finding is the gradual decrease in dopamine and serotonin levels during the study epochs, which may reflect a strain on the reward and mood regulation systems [23-25]. Dopamine, a key neurotransmitter in motivation and reward, is negatively affected by chronic stress, while decreased serotonin has been associated with depressive symptoms and emotional distress [6].

The positive correlation between physical exercise and levels of both dopamine and serotonin is consistent with previous studies documenting its beneficial effects on brain neurotransmitters [21]. In contrast, the negative correlation between screen time and dopamine suggests a possible dysregulation of the reward system through artificial overstimulation, as described in studies on "digital overexposure" [18].

A particularly interesting finding concerns the relationship between BMI and serotonin. Although some studies have reported a reduction in serotonin in individuals with increased body weight [26,27], others show increased activity of the serotonergic system, possibly due to compensatory mechanisms or the involvement of serotonin in the regulation of food intake and metabolism [28,29]. This relationship appears to be multifactorial and may vary depending on gender, age, metabolic profile, and microbiome status [28,30].

Strengths and limitations

Strengths include repeated measurements across five years, standardized morning sampling, and rigorous inclusion criteria that minimized confounding medical or pharmacological effects. The use of mixed-effects models allowed control for intra-individual variability and covariates. Nevertheless, limitations must be acknowledged. First, the study design is observational, which does not allow for causal conclusions to be drawn. Furthermore, the sample consists exclusively of university students, which limits the possibility of generalizing to other populations. Second, self-reports of screen time and physical activity may be subject to memory or subjectivity biases. Third, other potentially important factors, such as sleep quality, level of social support, diet, or inflammation markers, were not considered. Finally, the circadian rhythm of cortisol was not taken into account. However, all measurements of total and free cortisol were taken in the morning, when the concentration is at its peak. The consistent increase of these values does indeed indicate the effect that the pandemic had on the participants' stress mechanisms.

## Conclusions

This longitudinal study provides evidence that the COVID-19 pandemic and associated behavioral disruptions were correlated with prolonged activation of the HPA axis, as demonstrated by increased cortisol levels. The progressive reduction in dopamine and serotonin may reflect a disruption of reward and mood systems, confirming the effect of chronic stress in young adult university students. Increased screen exposure may be linked to the dysregulation of stress and reward mechanisms, while its association with free cortisol underlines the need for further study. On the other hand, physical exercise emerges as a protective factor with a positive effect on both cortisol and neurotransmitters. However, it should be noted that the correlation between BMI and serotonin levels suggests a more complex interaction between metabolic and neuropsychiatric parameters.

Future interventional studies should investigate whether physical exercise and the reduction of digital stimuli can restore disturbances in neuroendocrine and neurotransmitter function.
